# Associations of neutrophil-percentage-to-albumin ratio level with all-cause mortality and cardiovascular disease-cause mortality among patients with hypertension: evidence from NHANES 1999–2010

**DOI:** 10.3389/fcvm.2024.1397422

**Published:** 2024-07-17

**Authors:** Zhihao Liu, Lei Dong, Geng Shen, Yangyang Sun, Yuting Liu, Jiarong Mei, Jia Jia, Fangfang Fan, Wenye Wang, Wei Huang, Jianping Li

**Affiliations:** ^1^ Department of Cardiology, Peking University First Hospital, Beijing, China; ^2^ Institute of Cardiovascular Disease, Peking University First Hospital, Beijing, China; ^3^ State Key Laboratory of Vascular Homeostasis and Remodeling, Institute of Cardiovascular Sciences, School of Basic Medical Sciences, Peking University Health Science Center, Beijing, China; ^4^ NHC Key Laboratory of Cardiovascular Molecular Biology and Regulatory Peptides, Beijing, China

**Keywords:** neutrophil-percentage-to-albumin ratio, all-cause mortality, CVD-cause mortality, national health and nutrition examination survey, hypertension

## Abstract

**Background:**

The associations of neutrophil-percentage-to-albumin ratio (NPAR) level with all-cause and cardiovascular disease (CVD)-cause mortality among patients with hypertension remain unclear. This study aims to investigate the associations of NPAR level with all-cause and CVD-cause mortality among patients with hypertension.

**Methods:**

This prospective cohort study included 8,990 patients with hypertension who participated in the National Health and Nutrition Examination Survey (NHANES) from 1999 to 2010. Multivariable Cox proportional hazards regression models were used to compute hazard ratios and 95% CIs for the associations of NPAR level with all-cause mortality and CVD-cause mortality. Restricted cubic spline analyses were used to examine the nonlinear association of NPAR level with all-cause mortality and CVD-cause mortality.

**Results:**

This cohort study included data from 8,990 participants in analysis. During 104,474 person-years of follow-up, 3,069 all-cause deaths and 1,449 CVD-cause deaths were documented. Nonlinear associations were observed for NPAR levels with risk of all-cause mortality and CVD-cause mortality among patients with hypertension. Compared with participants in T1 of NPAR, there was a significantly increased risk of all-cause mortality and CVD-cause mortality for participants in both T2 and T3 in the fully adjusted model (model 3). The corresponding HRs for all-cause mortality were 1.10 (95% CI, 0.98–1.22) and 1.63 (95% CI, 1.45–1.82). The corresponding HRs for CVD-cause mortality were 1.10 (95% CI, 0.99–1.23) and 1.63 (95% CI, 1.46–1.81).

**Conclusions:**

Elevated NPAR level was significantly associated with an increased risk of all-cause and CVD-cause mortality in adults with hypertension. NPAR may be clinically useful for predicting long-term health outcomes and mortality in hypertensive population.

## Graphical Abstract

This prospective cohort study included 8,990 patients with hypertension who participated in the National Health and Nutrition Examination Survey (NHANES) from 1999 to 2010. In this large, prospective cohort study, we found significant nonlinear associations of NPAR level with all-cause and CVD-cause mortality. A high NPAR level was associated with a higher risk of all-cause and CVD-cause mortality.

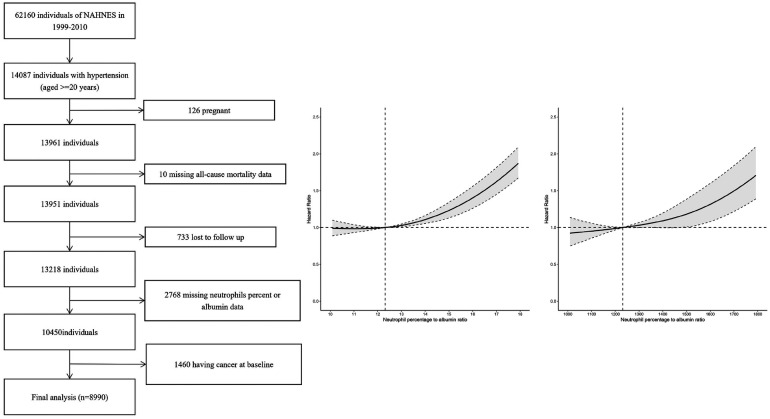

## Introduction

Hypertension is a significant risk factor for cardiovascular disease (CVD) and mortality, affecting approximately 30% of the global population and contributing to 14% of global deaths (1). In the United States, the prevalence of hypertension in adults varies between 32% and 46% (2). Epidemiological studies have indicated that more than half of individuals with hypertension do not undergo treatment, and less than a quarter successfully achieve normal blood pressure control through medication (3, 4). Consequently, the prognostic evaluation of hypertensive patients remains critical. The neutrophil-percentage-to-albumin ratio (NPAR) is an emerging biomarker of inflammation, derived from neutrophils and albumin (5). It is recognized for its accuracy in reflecting inflammation levels (6). Neutrophils, which are critical in the inflammatory response, typically exhibit elevated levels in various conditions (7). In contrast, albumin levels often show an inverse correlation with oxidative stress and inflammation (8). In addition, recent studies have demonstrated that the NPAR is a valuable prognostic biomarker for cardiogenic shock and myocardial infarction (9).

A secondary analysis of the canakinumab anti-inflammatory thrombosis outcome study (CANTOS) trial revealed heightened inflammation levels in individuals with severe hypertension (10). These findings emphasize a potentially crucial role of inflammation in the development of hypertension. Notably, the NPAR has shown significant associations with prognostic outcomes in patients with cardiovascular disease, stroke-associated infection, and pancreatic cancer (11–13). Furthermore, current literature suggests that there has been limited exploration of the association between NPAR level and mortality among hypertensive patients. To address these research gaps, we prospectively investigate the associations of NPAR level with risks of all-cause mortality and CVD-cause mortality in a nationally representative sample of US adults with hypertension.

## Methods

The National Health and Nutrition Examination Survey (NHANES), conducted periodically as a cross-sectional sampling survey. It is an initiative of the National Center for Health Statistics, which is a branch of the Centers for Disease Control and Prevention. This survey constitutes a nationally representative sample of the non-institutionalized civilian population of the United States. This study followed the Strengthening the Reporting of Observational Studies in Epidemiology (STROBE) reporting guidelines. The protocols of the NHANES study were approved by the Institutional Review Board of the National Center for Health Statistics. Informed written consent was obtained from all participants upon enrollment.

### Data availability

Comprehensive details regarding the data and materials have been made publicly available at the National Center for Health Statistics of the Centers for Disease Control and Prevention (https://www.cdc.gov/nchs/nhanes/index.htm).

### Sample

**Figure 1:**
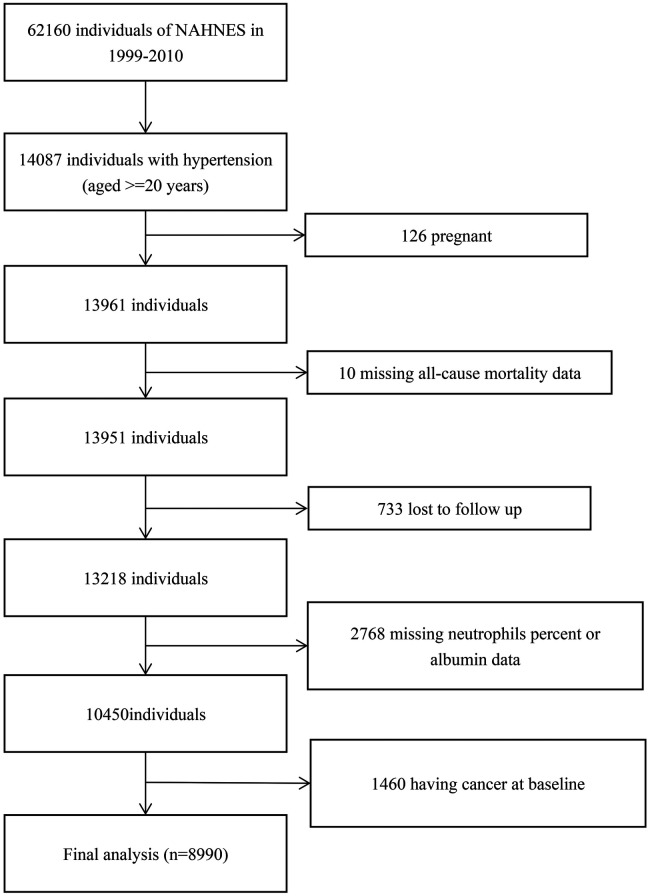
Study inclusion and exclusion flowchart.

In this cohort study, we included 14,087 participants with hypertension (≥20 years of age) of NHANES from 1999 through 2010. After excluding participants who were pregnant (*n* = 126), missed all-cause mortality data (*n* = 10), were lost to follow-up (*n* = 733), missed neutrophils percent or albumin data (*n* = 2,768), or had prevalent cancer (*n* = 1,460), 8,990 participants with hypertension were included (Figure 1).

### Definitions of hypertension

We defined hypertension based on the following three aspects: (1) Having been told or diagnosed with hypertension by a physician. (2) Use antihypertensive medication. (3) At least three blood pressure measurements showing an average systolic blood pressure (SBP) of ≥140 mmHg or diastolic blood pressure (DBP) of ≥90 mmHg.

### Clinical and demographic characteristics

In this study, we assessed race and ethnicity as fundamental demographic variables, categorizing them based on self-reported information during interviews. Body mass index (BMI) was computed as the ratio of weight in kilograms to the square of height in meters, and then categorized as lower than 28 or higher. Education level was also evaluated as a basic demographic variable, with classifications including under high school (less than 9th grade, 9–11th grade,12th grade with no diploma), high school (high school grad/GED or equivalent) and college or more (some college or AA degree, and college graduate or above). Poverty income ratio (PIR) was calculated as the ratio between family income and the poverty threshold. In this study, marital status was categorized into never married, married (married and living with partner) and unmarried (widowed, divorced, separated). Regarding smoking status, participants were sorted into three distinct groups: never smokers (never smoked or smoked less than 100 cigarettes in their lifetime and quit smoking), ever smokers (smoked more than 100 cigarettes but had subsequently quit), and current smokers (smoked more than 100 cigarettes and are presently smoking). The data pertaining to a physician-diagnosed history of diabetes, hypertension, and high cholesterol were gathered through self-reporting by the participants. Trained professionals, utilizing a drug supplement database, collected information on medications taken within the preceding 30 days by cross-referencing the products provided by the participants. In addition, levels of C-reactive protein (CRP), total cholesterol (TC), high-density lipoprotein cholesterol (HDL-C), and indicators of liver function [levels of alanine transaminase, lactate dehydrogenase, aspartate aminotransferase, gamma-glutamyl transpeptidase, and circulating homocysteine (only available in subsamples)] were measured at recruitment. We apply the Chronic Kidney Disease Epidemiology Collaboration (14) to calculate eGFR.

Blood samples were processed, frozen at −20°C, and then sent to the National Center for Environmental Health for comprehensive testing. A comprehensive description of the laboratory methods used can be found on the NHANES website. The methods used to derive the complete blood count are based on the Beckman Coulter methodology. The NPAR was calculated as the neutrophil percentage divided by the albumin using the same blood samples, according to the formula: (Neutrophilpercentage(%)×100/Albumin(g/dl)) (15). Plate count (PC), neutrophil count (NC), and lymphocyte count (LC) were measured in 1,000 cells/μl. Systemic immune-inflammation index (SII) was calculated as PC * (NC/LC). Detailed descriptions of the laboratory methodology are available at: https://wwwn.cdc.gov/Nchs/Nhanes/2003-2004/L25_C.html.

### Outcome measures

The acquisition of mortality data involved linking the cohort database with the National Death Index. All-cause mortality was defined as any documented cause of death. CVD mortality was defined employing the International Statistical Classification of Diseases and Related Health Problems, Tenth Revision (ICD-10). The specific codes utilized for this categorization included I00 to I09, I11, I13, I20 to I51, and I60 to I69.

### Statistical analysis

Considering the complex sampling design of the NHANES, all analyses incorporated sample weights, clustering, and stratification in the current study. The person-years for each participant were calculated from the date of recruitment until the date of death or the end of the follow-up period (December 31, 2019).

We performed weighted Kaplan–Meier curves with the log-rank tests for cumulative survival differences across different NPAR results. Multivariable Cox proportional hazards regression models were employed to derive hazard ratios (HRs) along with corresponding 95% confidence intervals (CIs) for assessing the associations of NPAR level with the risks of all-cause mortality and CVD-cause mortality. The Schoenfeld residuals were utilized to assess the assumption of proportional hazards, and no violation was detected.

Three multivariable models were constructed. Model 1 was a crude model that only neutrophil percentage to albumin ratio was incorporated. In model 2, we adjusted for age (continuous, years), sex (male or female), educational level (<high school, high school and ≥college), race and ethnicity (Mexican American, non-Hispanic Black, non-Hispanic White or other), PIR, marital status (never married, married and unmarried). In model 3, we additionally adjusted for smoking status (never, ever, or current), self-reported history of diabetes, hypertension or hypercholesterolemia, diabetes medication use (yes or no), lipid lowering drug (yes or no), BMI (continuous, kg/m^2^), SBP (mmHg) and DBP (mmHg).

Variables with missing data were imputed using the multiple imputation method, which was based on “mi” package. A restricted cubic spline analysis, utilizing four knots at the 5th, 35th, 65th, and 95th percentiles, was used to examine the nonlinear association of NPAR with all-cause mortality and CVD mortality. The 25th percentile was used as the reference point. This analysis was confined to values between the first and 95th percentiles to minimize the influence of potential outliers. Nonlinearity was tested using the likelihood ratio test. The associations of the tertiles of NPAR with mortality were examined using the first tertile as the reference group based on the results of restricted cubic spline analyses.

We further stratified the analyses by sex (male or female), age (<65 or ≥65 years), ethnicity (Mexican American, non-Hispanic Black, non-Hispanic White or other), educational level (<high school, high school, or ≥ college), smoking status (never, ever, or current), diabetes (yes or no), hypercholesterolemia (yes or no), BMI (<28 or ≥28 kg/m²), SBP (<140 or ≥140 mmHg), and DBP (<90 or ≥90 mmHg). The *P*-values for the product terms between NPAR level and the stratified factors were used to estimate the statistical significance of the interactions.

We also conducted a series of sensitivity analyses. (1) To minimize the potential for reverse causation bias, participants who died within 2 years of follow-up were excluded. (2) The main analyses were repeated across quintiles of NPAR level to assess consistency in the findings. (3) Participants with a prior history of CVD were further excluded from the main analyses. (4) To explore the possible role of inflammation, blood lipid levels, or liver and kidney function in the observed associations, we further adjusted for CRP levels, lipid profile (including TC, and HDL-C), an indicator of kidney function (estimated glomerular filtration rate), and indicators of liver function (levels of alanine transaminase, lactate dehydrogenase, aspartate aminotransferase, gamma-glutamyl transpeptidase).

All analyses were performed using R, version 4.3.2, and a 2-sided *P* < .05 was set as the threshold for statistical significance.

## Results

### Baseline characteristics of study participants

The weighted distribution of selected participants sociodemographic characteristics and other covariates according to NPAR tertiles is shown in Table 1. The ranges of NPAR for tertiles 1 through 3 were <12.9, 12.9–15.0, and ≥15.0. There were significant differences between NPAR tertiles for all included characteristics, except for self-reported high cholesterol and SBP. Compared with T1 and T2 of NPAR, participants in T3 were more likely to: be females, be a Non-Hispanic White, be married, have lower education level, have lower poverty income ratio, be a current smoker, have diabetes and use hypoglycemic drug, lipid lowering drug. Participants in T3 were more likely to have higher age, body mass index and lower DBP (all *P* < 0.05).

**Table 1:** Baseline characteristics of study participants.

Characteristics	Total (*N* = 8,990)	T1 (*N* = 2,993) (<12.9)	T2 (*N* = 3,000) (12.9–15.0)	T3 (*N* = 2,997) (≥15.0)	*P*-value
Sex, no. (%)					0.002
Male	4,480 (49.2)	1,557 (52)	1,500 (50)	1,423 (47.5)	
Female	4,510 (50.8)	1,436 (48)	1,500 (50)	1,574 (52.5)	
Age, mean (SE), y	59.8 (15.4)	57.7 (15)	59.8 ± 15.3	61.9 ± 15.7	<0.001
Ethnicity, no. (%)					<0.001
Mexican American	1,619 (5.6)	477 (15.9)	625 (20.8)	517 (17.3)	
Non-Hispanic White	4,378 (72.1)	1,243 (41.5)	1,538 (51.3)	1,597 (53.3)	
Non-Hispanic Black	2,079 (13.2)	937 (31.3)	561 (18.7)	581 (19.4)	
Others	914 (9.1)	336 (11.2)	276 (9.2)	302 (10.1)	
Education level, no. (%)					0.032
<High school	3,180 (22.8)	1,043 (34.8)	1,045 (34.8)	1,092 (36.4)	
High school	2,250 (28.1)	711 (23.8)	758 (25.3)	781 (26.1)	
College or more	3,560 (49.1)	1,239 (41.4)	1,197 (39.9)	1,124 (37.5)	
PIR, mean (SE)	2.5 (1.6)	2.5 (1.6)	2.5 (1.6)	2.3 (1.5)	<0.001
Marital status, no. (%)					<0.001
Never married	858 (9.9)	300 (10)	262 (8.7)	296 (9.9)	
Married	5,335 (64.6)	1,828 (61.1)	1,831 (61)	1,676 (55.9)	
Unmarried	2,797 (25.5)	865 (28.9)	907 (30.2)	1,025 (34.2)	
Smoking status, no. (%)					0.012
Never	4,460 (49.7)	1,558 (52.1)	1,476 (49.2)	1,426 (47.6)	
Ever	2,829 (30.9)	906 (30.3)	953 (31.8)	970 (32.4)	
Current	1,701 (19.4)	529 (17.7)	571 (19)	601 (20.1)	
Self-reported disease					
Diabetes, no. (%)	1,782 (14.8)	494 (16.5)	556 (18.5)	732 (24.4)	<0.001
High cholesterol	4,473 (50.1)	1,493 (49.9)	1,488 (49.6)	1,492 (49.8)	0.976
Hypoglycemic drug, no. (%)	1,558 (12.8)	416 (13.9)	502 (16.7)	640 (21.4)	<0.001
Lipid lowering drug, no. (%)	2,631 (28.0)	795 (26.6)	879 (29.3)	957 (31.9)	<0.001
BMI, median (IQR), kg/m^2^	29.4 (25.8, 34)	28.9 (25.6, 32.7)	29.4 (26, 33.7)	30.2 (25.9, 35.8)	<0.001
SBP, mean (SE), mmHg	136.8 (21.2)	137 (20.2)	136.7 (21.1)	136.6 (22.2)	0.792
DBP, mean (SE), mmHg	72.4 (15.9)	74.8 (15.1)	72.2 (16.2)	70.2 (16.2)	<0.001

PIR, Poverty income ratio; BMI, body mass index; SBP, systolic blood pressure; DBP, diastolic blood pressure; T1, tertiles 1; T2, tertiles 2; T3, tertiles3.

### Association of NPAR with all-cause mortality and CVD-cause mortality

We constructed 3 models for analyzing the independent role of NPAR in mortality. The HRs and 95% CI for these 3 equations are listed in Table 2. We converted NPAR from a continuous variable to a categorical variable (tertiles). Compared with participants in T1 of NPAR, there was a significantly increased risk of all-cause mortality and CVD-cause mortality for participants in both T2 and T3 in the fully adjusted model (model 3). The corresponding HRs for all-cause mortality were 1.10 (95% CI, 0.98–1.22) and 1.63 (95% CI, 1.45–1.82). The corresponding HRs for CVD-cause mortality were 1.10 (95% CI, 0.99–1.23) and 1.63 (95% CI, 1.46–1.81).

**Table 2:** Cox regression of the association between neutrophil percentage to albumin ratio and all-cause mortality and CVD-cause mortality among patients with hypertension.

Neutrophil percentage to albumin ratio	Person-y	No. of events	Mortality rate (per 1,000 person-y)	Model 1^a^		Model 2^b^		Model 3^c^	
				HR (95%CI)	*P*-value	HR (95%CI)	*P*-value	HR (95%CI)	*P*-value
All-cause mortality									
T1 (<12.9)	37,382	768	20.54	Ref.		Ref.		Ref.	
T2 (12.9–15.0)	36,046	974	27.02	1.24 (1.10, 1.40)	<0.001	1.14 (1.02, 1.27)	0.020	1.10 (0.98, 1.22)	0.100
T3 (≥15.0)	31,046	1,327	42.74	2.16 (1.94, 2.40)	<0.001	1.70 (1.52, 1.91)	<0.001	1.63 (1.45, 1.82)	<0.001
*P* for trend					<0.001		<0.001		<0.001
CVD-cause mortality									
T1 (<12.9)	37,382	920	24.61	Ref.		Ref.		Ref.	
T2 (12.9–15.0)	36,046	228	6.33	1.33 (1.12, 1.57)	<0.001	1.20 (1.01, 1.43)	0.040	1.10 (0.99, 1.23)	0.088
T3 (≥15.0)	31,046	301	9.70	2.23 (1.85, 2.68)	<0.001	1.69 (1.42, 2.02)	<0.001	1.63 (1.46, 1.81)	<0.001
*P* for trend					<0.001		<0.001		<0.001

CVD, cardiovascular disease; CI, confidence interval; HR, hazard ratio; T1, tertiles 1; T2, tertiles 2; T3, tertiles3.

^a^ Model 1 was a crude model that only incorporates neutrophil percentage to albumin ratio.

^b^ Model 2 adjusted for age, sex, ethnicity, education level, poverty income ratio and marital status.

^c^ Model 3 adjusted for age, sex, ethnicity, education level, poverty income ratio, marital status, smoking status, self-reported diabetes, self-reported high cholesterol, hypoglycemic drug, lipid lowering drug, body mass index, systolic blood pressure, diastolic blood pressure.

### Nonlinear association of NPAR with all-cause mortality and CVD-cause mortality

**Figure 2:**
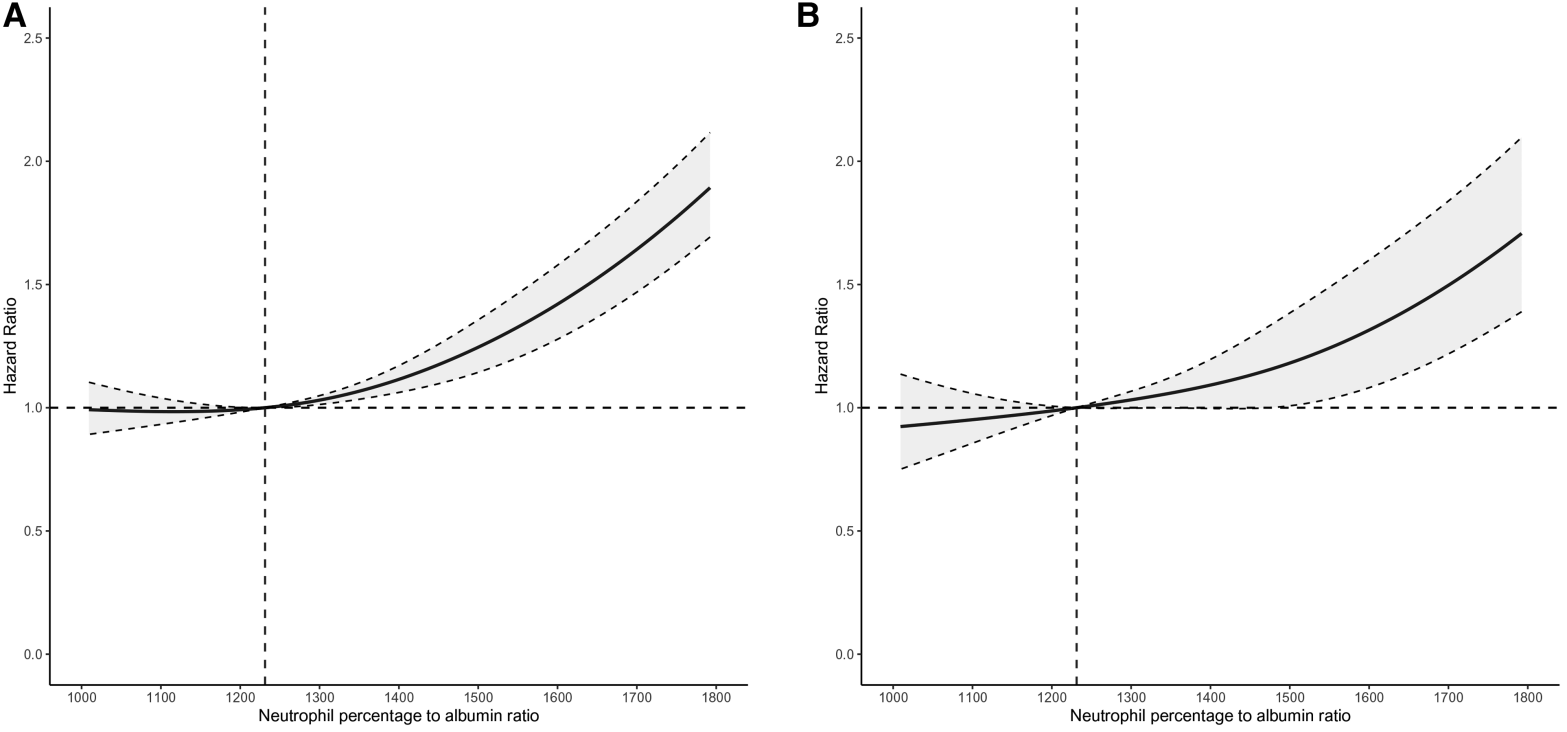
Dose-response relationships of NPAR level with the probability of all-cause mortality (**A**) and CVD-cause mortality (**B**). A nonlinear association of NPAR level with both all-cause and CVD-cause mortality was found (*P* < 0.001). The solid line and dashed line represent the estimated values and their corresponding 95% confidence intervals. Adjustment factors included age, sex, ethnicity, education level, poverty income ratio, marital status, smoking status, self-reported diabetes, self-reported high cholesterol, hypoglycemic drug, lipid lowering drug, body mass index, systolic blood pressure and diastolic blood pressure.

To address the nonlinearity of the relation between NPAR and mortality, a smooth curve fitting (penalized spline method) was conducted. The fully adjusted smooth curve fitting showed a nonlinear association of NPAR with all-cause mortality (Figure 2A) and CVD-cause mortality (Figure 2B).

### Survival analysis

**Figure 3:**
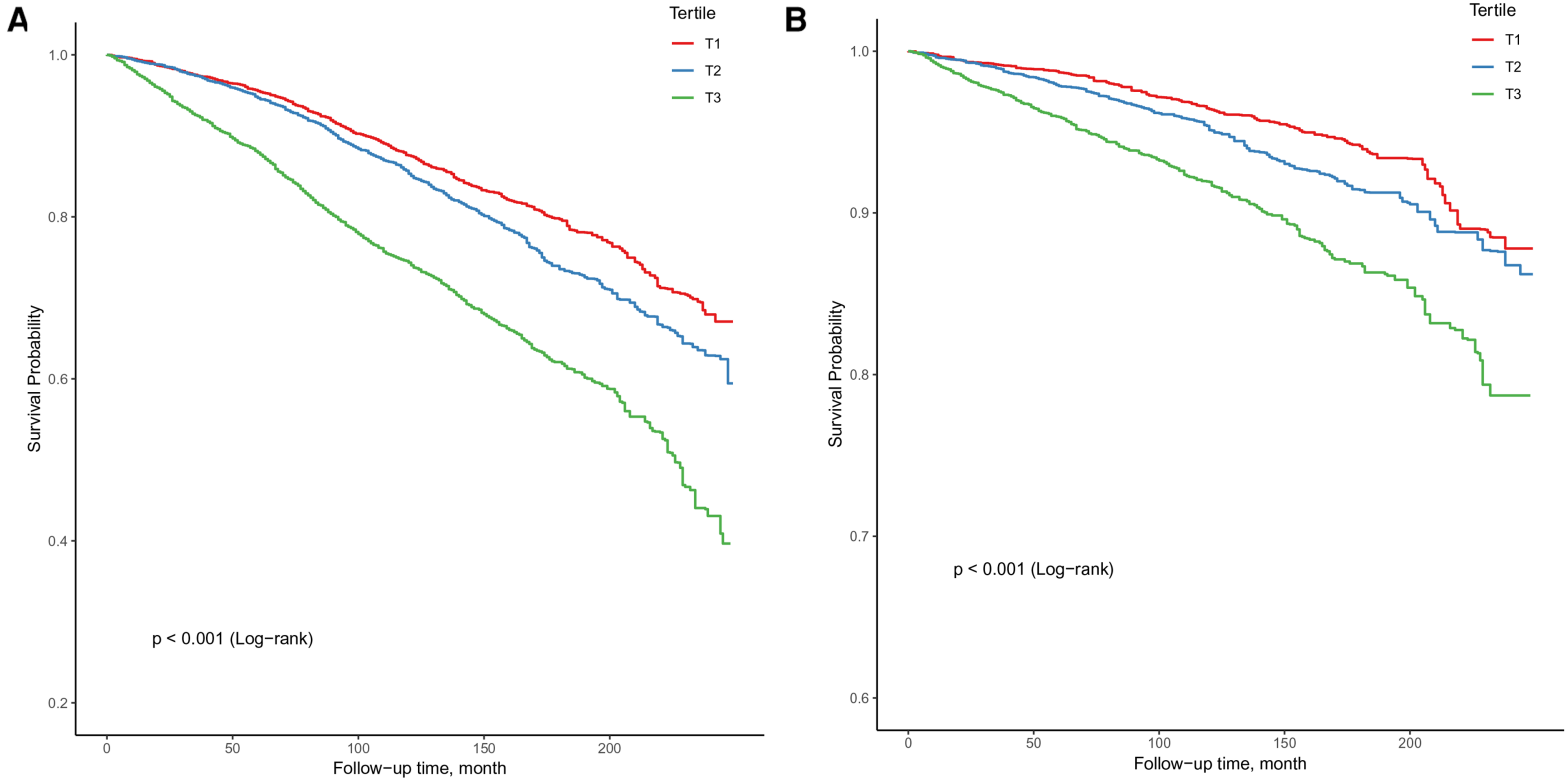
Kaplan-Meier curves for survival probability, with follow-up in years. (**A**) All-cause mortality; (**B**) CVD-cause mortality.

For the analysis of NPAR level (8,990 adults; mean (SE) age, 59.8 (15.4) years; 4,510 women (weighted, 50.8%) and 4,480 men (weighted, 49.2%)), we identified 3,069 all-cause deaths and 1,449 CVD-cause deaths during 104,474 person-years of follow-up. Figure 3 illustrates Kaplan-Meier curves for survival probability at the start of follow-up, for which unadjusted log-rank tests showed that all-cause mortality (Figure 3A**)** and CVD-cause mortality (Figure 3B**)** were significantly elevated in T3 in comparison with T1 and T2 (*P* < 0.001).

### Comparison of NPAR with systemic immune inflammation index (SII) and segmented neutrophils percent models

**Figure 4:**
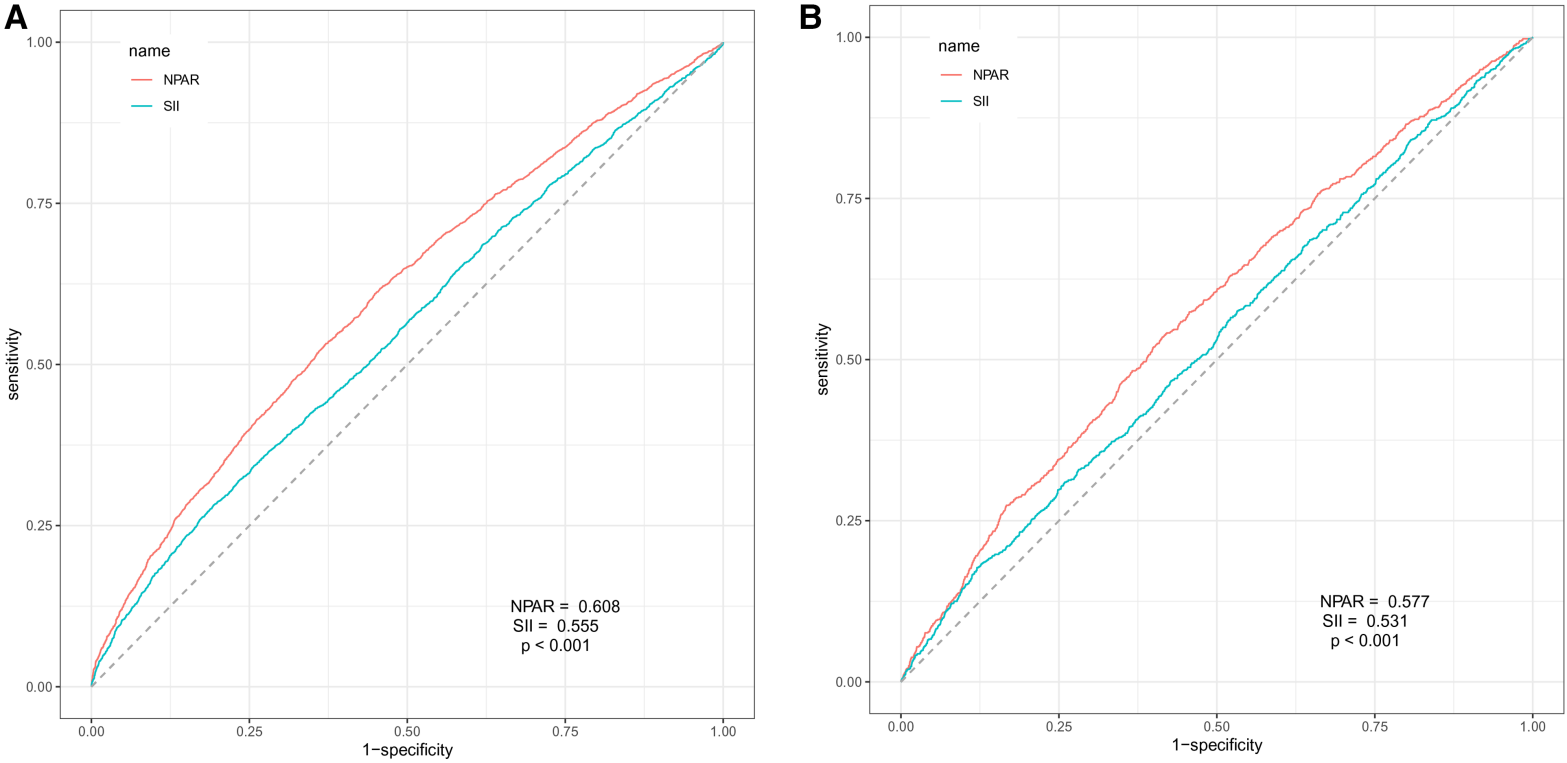
ROC curve analysis for NPAR and SII prediction of all-cause mortality (**A**) and CVD-cause mortality (**B**) in the hypertensive population.

The predictive value of NPAR for all-cause mortality (AUC: 0.608 vs. 0.555; *P* < 0.001; Figure 4A) and CVD-cause mortality (AUC: 0.577 vs. 0.531; *P* < 0.001; Figure 4B) in the overall hypertensive population is superior to that of SII. Moreover, the predictive value of NPAR for all-cause mortality (AUC: 0.608 vs. 0.587; *P* < 0.001; Supplementary Figure S5A) and CVD-cause mortality (AUC: 0.577 vs. 0.561; *P* = 0.001; Supplementary Figure S5B) in the hypertensive population is superior to segmented neutrophils percent.

### Stratified and sensitivity analyses

We found a significant interaction between NPAR level and age with the risk of all-cause mortality (*P* = .00 for interaction). For the subgroup aged <65 compared with the reference group (T1), the HR of all-cause mortality was 1.68 (95% CI, 1.38–2.06) in T3, and in the subgroup aged ≥65 compared with the reference group, the HR of all-cause mortality was 1.69 (95% CI, 1.50–.90) in T3. However, no significant interactions were found between NPAR level and any other strata variables with the risk of all-cause mortality and CVD-cause mortality after correcting for multiple testing (Table 3).

**Table 3:** Subgroup analysis of the association of neutrophil percentage to albumin ratio with all-cause mortality and CVD-cause mortality.

	All-cause mortality				CVD-cause mortality			
Subgroups	Hazard ratio (95% CIs) by tertile			*P* for interaction	Hazard ratio (95% CIs) by tertile			*P* for interaction
	T1	T2	T3		T1	T2	T3	
Sex				0.411				0.254
Male	Ref.	1.00 (0.85, 1.18)	1.56 (1.30, 1.87)		Ref.	1.11 (0.83, 1.48)	1.27 (0.93, 1.74)	
Female	Ref.	1.19 (1.02, 1.39)	1.69 (1.45, 1.96)		Ref.	1.16 (0.90, 1.48)	1.86 (1.47, 2.34)	
Age, year				<0.001				0.377
<65	Ref.	0.90 (0.71, 1.15)	1.68 (1.38, 2.06)		Ref.	1.02 (0.68, 1.52)	1.58 (1.01, 2.45)	
≥65	Ref.	1.27 (1.10, 1.48)	1.69 (1.50, 1.90)		Ref.	1.23 (0.97, 1.56)	1.59 (1.29, 1.94)	
Ethnicity				0.187				0.810
Mexican American	Ref.	1.01 (0.72, 1.42)	1.80 (1.28, 2.54)		Ref.	1.29 (0.70, 2.38)	1.54 (0.80, 2.95)	
Non-Hispanic White	Ref.	1.07 (0.93, 1.22)	1.50 (1.31, 1.71)		Ref.	1.13 (0.91, 1.41)	1.48 (1.16, 1.87)	
Non-Hispanic Black	Ref.	1.19 (0.91, 1.56)	2.15 (1.78, 2.60)		Ref.	1.24 (0.83, 1.85)	1.85 (1.28, 2.67)	
Others	Ref.	1.08 (0.65, 1.80)	2.14 (1.53, 2.99)		Ref.	0.80 (0.28, 2.23)	1.80 (0.80, 4.05)	
Education level				0.196				0.122
<High school	Ref.	1.04 (0.87, 1.24)	1.47 (1.19, 1.81)		Ref.	1.21 (0.90, 1.63)	1.18 (0.82, 1.71)	
High school	Ref.	1.28 (1.02, 1.62)	2.09 (1.64, 2.66)		Ref.	1.05 (0.65, 1.71)	1.79 (1.23, 2.61)	
College or more	Ref.	1.03 (0.82, 1.29)	1.48 (1.21, 1.80)		Ref.	1.11 (0.76, 1.62)	1.82 (1.28, 2.59)	
Smoking status				0.500				0.418
Never	Ref.	1.15 (0.99, 1.33)	1.57 (1.32, 1.86)		Ref.	1.33 (1.04, 1.71)	1.50 (1.12, 2.02)	
Ever	Ref.	1.09 (0.88, 1.36)	1.77 (1.47, 2.14)		Ref.	0.91 (0.59, 1.41)	1.42 (1.01, 2.00)	
Current	Ref.	1.00 (0.75, 1.34)	1.41 (1.04, 1.90)		Ref.	1.06 (0.66, 1.71)	1.59 (0.96, 2.63)	
Diabetes				0.333				0.402
No	Ref.	1.13 (1.01, 1.27)	1.60 (1.40, 1.82)		Ref.	1.24 (1.01, 1.54)	1.59 (1.30, 1.94)	
Yes	Ref.	0.97 (0.75, 1.24)	1.65 (1.29, 2.10)		Ref.	0.96 (0.67, 1.38)	1.46 (0.91, 2.34)	
High cholesterol				0.220				0.067
No	Ref.	1.09 (0.92, 1.28)	1.75 (1.48, 2.06)		Ref.	1.20 (0.92, 1.57)	1.81 (1.35, 2.41)	
Yes	Ref.	1.10 (0.94, 1.28)	1.51 (1.29, 1.78)		Ref.	1.10 (0.84, 1.44)	1.35 (1.04, 1.77)	
BMI, kg/m2				0.881				0.162
<30	Ref.	1.12 (0.98, 1.28)	1.57 (1.36, 1.80)		Ref.	0.98 (0.78, 1.24)	1.46 (1.15, 1.84)	
≥30	Ref.	1.02 (0.80, 1.31)	1.59 (1.32, 1.92)		Ref.	1.39 (0.95, 2.04)	1.69 (1.24, 2.30)	
SBP, mmHg				0.996				0.782
<140	Ref.	1.09 (0.90, 1.31)	1.62 (1.36, 1.93)		Ref.	1.07 (0.81, 1.42)	1.51 (1.14, 2.02)	
≥140	Ref.	1.12 (0.95, 1.33)	1.64 (1.42, 1.90)		Ref.	1.22 (0.95, 1.56)	1.63 (1.27, 2.10)	
DBP, mmHg				0.674				0.506
<90	Ref.	1.10 (0.99, 1.23)	1.61 (1.43, 1.82)		Ref.	1.14 (0.95, 1.38)	1.51 (1.25, 1.82)	
≥90	Ref.	1.01 (0.65, 1.55)	1.92 (1.27, 2.92)		Ref.	1.02 (0.52, 1.99)	2.77 (1.46, 5.27)	

BMI, body mass index (calculated as weight in kilograms divided by height in meters squared); CVD, cardiovascular disease.

NHANES, National Health and Nutrition Examination Survey.

Model adjusted for age, sex, ethnicity, education level, poverty income ratio, marital status, smoking status, self-reported diabetes, self-reported high cholesterol, hypoglycemic drug, lipid lowering drug, body mass index, systolic blood pressure, diastolic blood pressure. The strata variable was not included when stratifying by itself.

CI, confidence interval; HR, hazard ratio; T1, tertiles 1; T2, tertiles 2; T3, tertiles3.

The results were generally robust in sensitivity analyses when excluding the participants who died within 2 years of follow-up (Supplementary Table S1), excluding participants who had a history of CVD at baseline (Supplementary Table S2), repeating the main analyses by quintiles of NPAR level (Supplementary Table S3). The associations remained substantially unchanged upon further adjustment for CRP levels, lipid levels, or liver function–related or kidney function–related indicators (models 2, 3, 4, and 5; Supplementary Table S4), including participants whose systolic blood pressure (SBP) ≥130 mmHg or diastolic blood pressure (DBP) ≥80 mmHg (Supplementary Table S5).

## Discussion

In this large, prospective cohort study of US adults with hypertension, we found significant nonlinear associations of NPAR level with all-cause and CVD-cause mortality. A high NPAR level was associated with a higher risk of all-cause and CVD-cause mortality. A variety of stratified analyses and sensitivity analyses indicated the robustness of our findings. Additionally, we compared the discrimination of the NPAR and traditional models in predicting all-cause and CVD-cause mortality in the entire hypertensive population. The results showed that the NPAR was better.

The relationship between NPAR level and mortality has been studied in various populations. NPAR levels were significantly higher in patients who died within 1-year post-surgery. Chou-Chin Lan et al. (16) conducted an analysis of 1,158 subjects diagnosed with chronic obstructive pulmonary disease (COPD) using data from the NHANES database spanning the years 2011–2018. The results demonstrated a significant association between increased NPAR and higher risks of all-cause and CVD-cause mortality. Notably, NPAR exhibited superior predictive accuracy for 5-year all-cause mortality compared to other hematologic inflammatory biomarkers, including the neutrophil-to-lymphocyte ratio (NLR), eosinophil-to-lymphocyte ratio (ELR), and similar markers. In the study by Jiasheng Cai et al. (17) involving 1,141 patients aged 80 years and older diagnosed with atrial fibrillation (AF), the level of NPAR exhibited a significant association with 28-day all-cause mortality after adjusting for confounding variables. In patients undergoing peritoneal dialysis (PD), Youqun Gao et al.'s (18) study revealed that individuals with elevated NPAR are associated with a higher risk of mortality. Although these studies consistently demonstrate a strong correlation between NPAR level and the risk of all-cause or CVD-cause mortality, it is important to note that research exploring this association within the hypertensive population is limited, with some studies suffering from small sample sizes. In the present study with a larger sample size, we found nonlinear associations of NPAR level with all-cause and CVD-cause mortality among 8,990 US adults with hypertension. High NPAR level was significantly associated with an increased risk of all-cause and CVD-cause mortality after adjusting for potential confounders.

The underlying mechanism linking NPAR level to mortality risk remains unclear. Recent evidence increasingly emphasizes the critical role of abnormal immune activation and inflammatory responses in the pathogenesis of hypertension (19). In murine models of hypertension, there is a heightened enhancement of antigen-specific T-cell immune responses, with T-cell-mediated inflammation playing a crucial role in the progression of hypertension (20). NPAR, a recently identified biomarker, indicates systemic infection and inflammation (12). NPAR combines the percentage of neutrophils and albumin levels, integrating various factors related to inflammation and immune response (21). Changes in NPAR can reflect the dynamic balance of immunity, inflammation and activity of diseases. Neutrophils, as essential components of the innate immune system, play a critical role in mediating inflammatory responses (22). During inflammation, activated neutrophils release reactive oxygen species (ROS), chemokines, and NADPH oxidase, leading to oxidative stress and impairing endothelial function (23–25). Neutrophils contribute to endothelial damage, thereby promoting atherosclerosis and thrombosis in hypertensive patients, which could potentially impact their prognosis (26). Studies suggest that lower levels of albumin are associated with an increased risk of mortality from cardiovascular disease (27). The principal mechanisms may involve the anti-inflammatory and antioxidant properties of albumin. It functions to inhibit pro-inflammatory cytokines by modulating interactions between neutrophils and endothelial cells, thereby leading to anticoagulant and anti-platelet aggregation activities (28). Furthermore, hypoalbuminemia reduces the effectiveness of the immune system, increasing susceptibility to infections. Low serum albumin levels also indicate malnutrition in patients (29). More research on the mechanisms of NPAR is needed in the future.

### Strengths and limitations

Our study has some strengths. To our knowledge, the present study is the largest investigation of the associations of NPAR level with all-cause and CVD-cause mortality among individuals with hypertension using restricted cubic spline analysis, with consideration of a multitude of potential confounding factors. NPAR is easy to calculate, cost-effective, and can be implemented even in areas with poor medical care. In addition, the present analysis is based on a nationally representative sample of US adults with hypertension, which facilitates the generalization of the findings.

Potential limitations of our study should also be noted. First, this study is observational, and a prospective multicenter study is needed to further confirm our conclusions. Second, because certain sections of the NHANES surveys depend on individual interviews and questionnaires, there may be inaccuracies in reporting or recall bias. Third, the circulating NPAR level were based on a single serum measurement, which may not accurately reflect the long-term status.

## Conclusions

In conclusion, our results from a large, nationally representative, and prospective study indicated that elevated NPAR level was significantly associated with an increased risk of all-cause and CVD-cause mortality in adults with hypertension. Our findings support the hypothesis that NPAR may be clinically useful for predicting long-term health outcomes and mortality in hypertensive population.

## Data Availability

Publicly available datasets were analyzed in this study. This data can be found here: https://doi.org/10.5061/dryad.d5h62.
