# Supplementary material for: Associations of neutrophil-percentage-to-albumin ratio level with all-cause mortality and cardiovascular disease-cause mortality among patients with hypertension: evidence from NHANES 1999–2010
Source: Front Cardiovasc Med. 2024 Jul 17;11:1397422. doi: 10.3389/fcvm.2024.1397422 (PMC11288876; doi:10.3389/fcvm.2024.1397422)

Supplementary Table 1. Cox regression of the association between neutrophil percentage to albumin ratio and all-cause mortality and CVD-cause mortality among patients with hypertension with excluding individuals with less than 2 years of follow-up

**Neutrophil percentage to albumin ratio**	**Person-y**	**No. of Events**	**Mortality Rate (per 1000 Person-y)**	**Model 1***		**Model 2#**		**Model 3†**	
				**HR (95%CI)**	***P*-value**	**HR (95%CI)**	***P*-value**	**HR (95%CI)**	***P*-value**
**All-cause mortality**									
T 1 (<12.9)	36659	697	19.01	Ref.		Ref.		Ref.	
T 2 (12.9-14.9)	35540	888	24.99	1.25 (1.10, 1.42)	<0.001	1.16 (1.03, 1.30)	0.014	1.11 (0.99, 1.25)	0.067
T 3 (≥14.9)	31927	1156	36.21	2.04 (1.81, 2.29)	<0.001	1.64 (1.45, 1.84)	<0.001	1.56 (1.38, 1.75)	<0.001
P for trend					<0.001		<0.001		<0.001
**CVD-cause mortality**									
T 1 (<12.9)	93665	197	2.10	Ref.		Ref.		Ref.	
T 2 (12.9-14.9)	91282	273	2.99	1.40 (1.16, 1.69)	<0.001	1.28 (1.06, 1.55)	0.012	1.11 (0.99, 1.25)	0.067
T 3 (≥14.9)	84635	329	3.89	2.17 (1.76, 2.69)	<0.001	1.69 (1.40, 2.05)	<0.001	1.56 (1.39, 1.76)	<0.001
P for trend					<0.001		<0.001		<0.001

*Model 1 was a crude model that only incorporates neutrophil percentage to albumin ratio.

#Model 2 adjusted for age, sex, ethnicity, education level, family poverty income ratio and marital status.

†Model 3 adjusted for age, sex, ethnicity, education level, family poverty income ratio, marital status, smoking status, self-reported diabetes, self-reported high cholesterol, hypoglycemic drug, lipid lowering drug, body mass index, systolic blood pressure, diastolic blood pressure.

Abbreviation: CVD, cardiovascular disease; CI, confidence interval; HR, hazard ratio.

Supplementary Table 2. Cox regression of the association between neutrophil percentage to albumin ratio and all-cause mortality and CVD-cause mortality excluding participants who had CVD history at baseline

Neutrophil percentage to albumin ratio	Person-y	No. of Events	Mortality Rate (per 1000 Person-y)	Model 1*		Model 2#		Model 3†	
				HR (95%CI)	*P*-value	HR (95%CI)	*P*-value	HR (95%CI)	*P*-value
**All-cause mortality**									
T 1 (<12.9)	30240	499	16.50	Ref.		Ref.		Ref.	
T 2 (12.9-15.1)	29560	601	20.33	1.17 (0.99, 1.38)	0.058	1.08 (0.94, 1.25)	0.280	1.07 (0.94, 1.23)	0.310
T 3 (≥15.1)	26539	817	30.78	1.94 (1.69, 2.22)	<0.001	1.59 (1.38, 1.84)	<0.001	1.55 (1.35, 1.78)	<0.001
P for trend					<0.001		<0.001		<0.001
**CVD-cause mortality**									
T 1 (<12.9)	30240	134	4.43	Ref.		Ref.		Ref.	
T 2 (12.9-15.1)	29560	160	5.41	1.25 (0.97, 1.61)	0.079	1.16 (0.91, 1.49)	0.230	1.07 (0.93, 1.22)	0.360
T 3 (≥15.1)	26539	222	8.37	2.30 (1.78, 2.98)	<0.001	1.84 (1.44, 2.34)	<0.001	1.55 (1.35, 1.77)	<0.001
P for trend					<0.001		<0.001		<0.001

*Model 1 was a crude model that only incorporates neutrophil percentage to albumin ratio.

#Model 2 adjusted for age, sex, ethnicity, education level, family poverty income ratio and marital status.

†Model 3 adjusted for age, sex, ethnicity, education level, family poverty income ratio, marital status, smoking status, self-reported diabetes, self-reported high cholesterol, hypoglycemic drug, lipid lowering drug, body mass index, systolic blood pressure, diastolic blood pressure.

Abbreviation: CVD, cardiovascular disease; CI, confidence interval; HR, hazard ratio.

Supplementary Table 3. Cox regression of the association between quintiles of neutrophil percentage to albumin ratio and all-cause mortality and CVD-cause mortality

Neutrophil percentage to albumin ratio	Person-y	No. of Events	Mortality Rate (per 1000 Person-y)	Model 1*		Model 2#		Model 3†	
				HR (95%CI)	*P*-value	HR (95%CI)	*P*-value	HR (95%CI)	*P*-value
**All-cause mortality**									
Quintiles 1 (<11.9)	22825	446	19.54	Ref.		Ref.		Ref.	
Quintiles 2 (11.9-13.3)	21990	487	22.15	1.12 (0.95, 1.31)	0.170	1.12 (0.95, 1.33)	0.180	1.12 (0.95, 1.32)	0.180
Quintiles 3 (13.3-14.6)	21616	590	27.29	1.31 (1.13, 1.51)	<0.001	1.16 (1.01, 1.34)	0.035	1.12 (0.97, 1.29)	0.110
Quintiles 4 (14.6-16.1)	20387	668	32.77	1.76 (1.50, 2.05)	<0.001	1.44 (1.23, 1.68)	<0.001	1.41 (1.20, 1.65)	<0.001
Quintiles 5 (≥ 16.1)	17655	878	49.73	2.57 (2.24, 2.96)	<0.001	2.05 (1.75, 2.41)	<0.001	1.92 (1.64, 2.25)	<0.001
P for trend					<0.001		<0.001		<0.001
**CVD-cause mortality**									
Quintiles 1 (<11.9)	57365	133	2.32	Ref.		Ref.		Ref.	
Quintiles 2 (11.9-13.3)	56015	150	2.68	1.14 (0.87, 1.49)	0.330	1.15 (0.88, 1.49)	0.300	1.11 (0.95, 1.31)	0.200
Quintiles 3 (13.3-14.6)	55344	176	3.18	1.34 (1.07, 1.69)	0.012	1.18 (0.93, 1.49)	0.180	1.12 (0.97, 1.28)	0.120
Quintiles 4 (14.6-16.1)	53032	197	3.71	1.73 (1.32, 2.27)	<0.001	1.38 (1.05, 1.80)	0.019	1.40 (1.19, 1.65)	<0.001
Quintiles 5 (≥ 16.1)	48257	264	5.47	2.77 (2.18, 3.54)	<0.001	2.12 (1.67, 2.70)	<0.001	1.91 (1.63, 2.23)	<0.001
P for trend					<0.001		<0.001		<0.001

*Model 1 was a crude model that only incorporates neutrophil percentage to albumin ratio.

#Model 2 adjusted for age, sex, ethnicity, education level, family poverty income ratio and marital status.

†Model 3 adjusted for age, sex, ethnicity, education level, family poverty income ratio, marital status, smoking status, self-reported diabetes, self-reported high cholesterol, hypoglycemic drug, lipid lowering drug, body mass index, systolic blood pressure, diastolic blood pressure.

Abbreviation: CVD, cardiovascular disease; CI, confidence interval; HR, hazard ratio.

Supplementary Table 4. Cox analysis of the association between neutrophil percentage to albumin ratio and all-cause mortality among patients with hypertension with further adjustment of several biomarkers

	Hazard ratio (95% CIs) by tertile			*P* for trend
	T 1	T 2	T 3	
**All-cause mortality**				
Model 1	Ref.	1.10 (0.98, 1.22)	1.63 (1.45, 1.82)	<0.001
Model 2	Ref.	1.09 (0.98, 1.22)	1.58 (1.41, 1.76)	<0.001
Model 3	Ref.	1.09 (0.98, 1.22)	1.61 (1.44, 1.80)	<0.001
Model 4	Ref.	1.08 (0.96, 1.20)	1.60 (1.43, 1.79)	<0.001
Model 5	Ref.	1.09 (0.98, 1.21)	1.59 (1.43, 1.78)	<0.001
**CVD-cause mortality**				
Model 1	Ref.	1.10 (0.99, 1.23)	1.63 (1.46, 1.81)	<0.001
Model 2	Ref.	1.10 (0.98, 1.22)	1.58 (1.42, 1.76)	<0.001
Model 3	Ref.	1.10 (0.98, 1.22)	1.61 (1.44, 1.80)	<0.001
Model 4	Ref.	1.08 (0.97, 1.21)	1.60 (1.43, 1.79)	<0.001
Model 5	Ref.	1.09 (0.98, 1.22)	1.60 (1.43, 1.78)	<0.001

Abbreviations: BMI, body mass index (calculated as weight in kilograms divided by height in meters squared); CVD, cardiovascular disease;

NHANES, National Health and Nutrition Examination Survey.

Model 1 adjusted for age, sex, ethnicity, education level, family poverty income ratio, marital status, smoking status, self-reported diabetes, self-reported high cholesterol, hypoglycemic drug, lipid lowering drug, body mass index, systolic blood pressure, diastolic blood pressure.

Model 2: Model 1 + CRP

Model 3: Model 1 + TC and HDL

Model 4: Model 1 + AST, ALT, GGT, LDH

Model 5: Model 1 + eGFR

Abbreviation: CI, confidence interval; HR, hazard ratio.

Supplementary Table 5. Cox regression of the association between the neutrophil-to-albumin ratio and all-cause mortality, as well as cardiovascular disease (CVD)-related mortality, in patients with hypertension, according to the diagnostic criteria set forth by the 2017 American Heart Association (AHA) guidelines.

**Neutrophil percentage to albumin ratio**	**Person-y**	**No. of Events**	**Mortality Rate (per 1000 Person-y)**	**Model 1***		**Model 2#**		**Model 3†**	
				**HR (95%CI)**	***P*-value**	**HR (95%CI)**	***P*-value**	**HR (95%CI)**	***P*-value**
**All-cause mortality**									
T 1 (<12.77)	48125	816	16.96	ref		ref		ref	
T 2 (12.77-14.86)	46486	1022	21.99	1.24 (1.09, 1.41)	<0.001	1.10 (0.98, 1.23)	0.11	1.05 (0.94, 1.17)	0.36
T 3 (≥14.86)	40172	1487	37.02	2.25 (2.01, 2.51)	<0.001	1.62 (1.47, 1.79)	<0.001	1.54 (1.40, 1.69)	<0.001
P for trend					<0.001		<0.001		<0.001
**CVD-cause mortality**									
T 1 (<12.77)	48125	231	4.80	ref		ref		ref	
T 2 (12.77-14.86)	46486	301	6.48	1.35 (1.09, 1.66)	0.005	1.17 (0.96, 1.43)	0.13	1.11 (0.91, 1.34)	0.32
T 3 (≥14.86)	40172	443	11.03	2.55 (2.13, 3.06)	<0.001	1.75 (1.47, 2.07)	<0.001	1.58 (1.33, 1.89)	<0.001
P for trend					<0.001		<0.001		<0.001

*Model 1 was a crude model that only incorporates neutrophil percentage to albumin ratio.

#Model 2 adjusted for age, sex, ethnicity, education level, family poverty income ratio and marital status.

†Model 3 adjusted for age, sex, ethnicity, education level, family poverty income ratio, marital status, smoking status, self-reported diabetes, self-reported high cholesterol, hypoglycemic drug, lipid lowering drug, body mass index, systolic blood pressure, diastolic blood pressure.

Abbreviation: CVD, cardiovascular disease; CI, confidence interval; HR, hazard ratio.

Supplementary Figure 1A

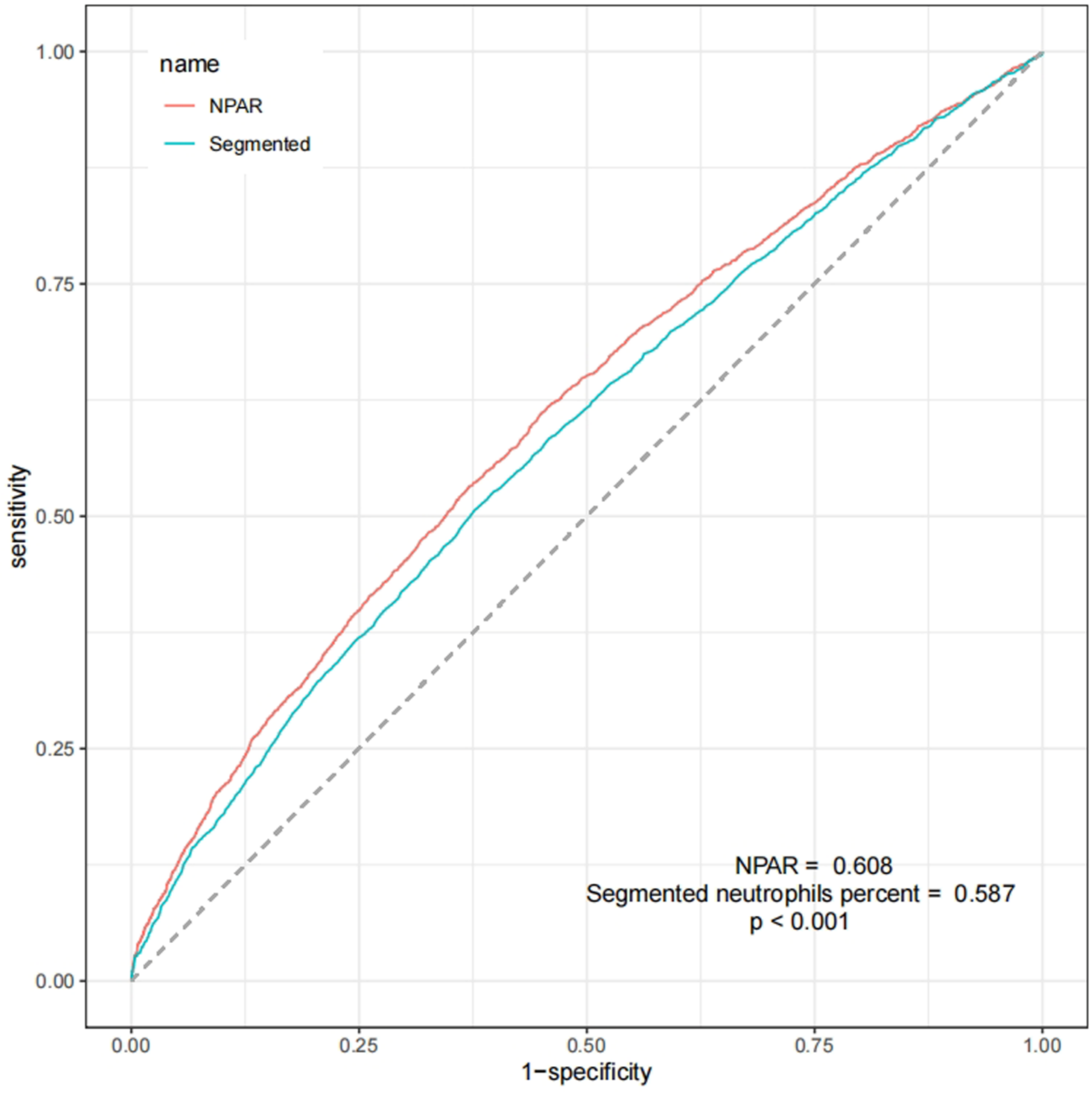

Supplementary Figure 1B

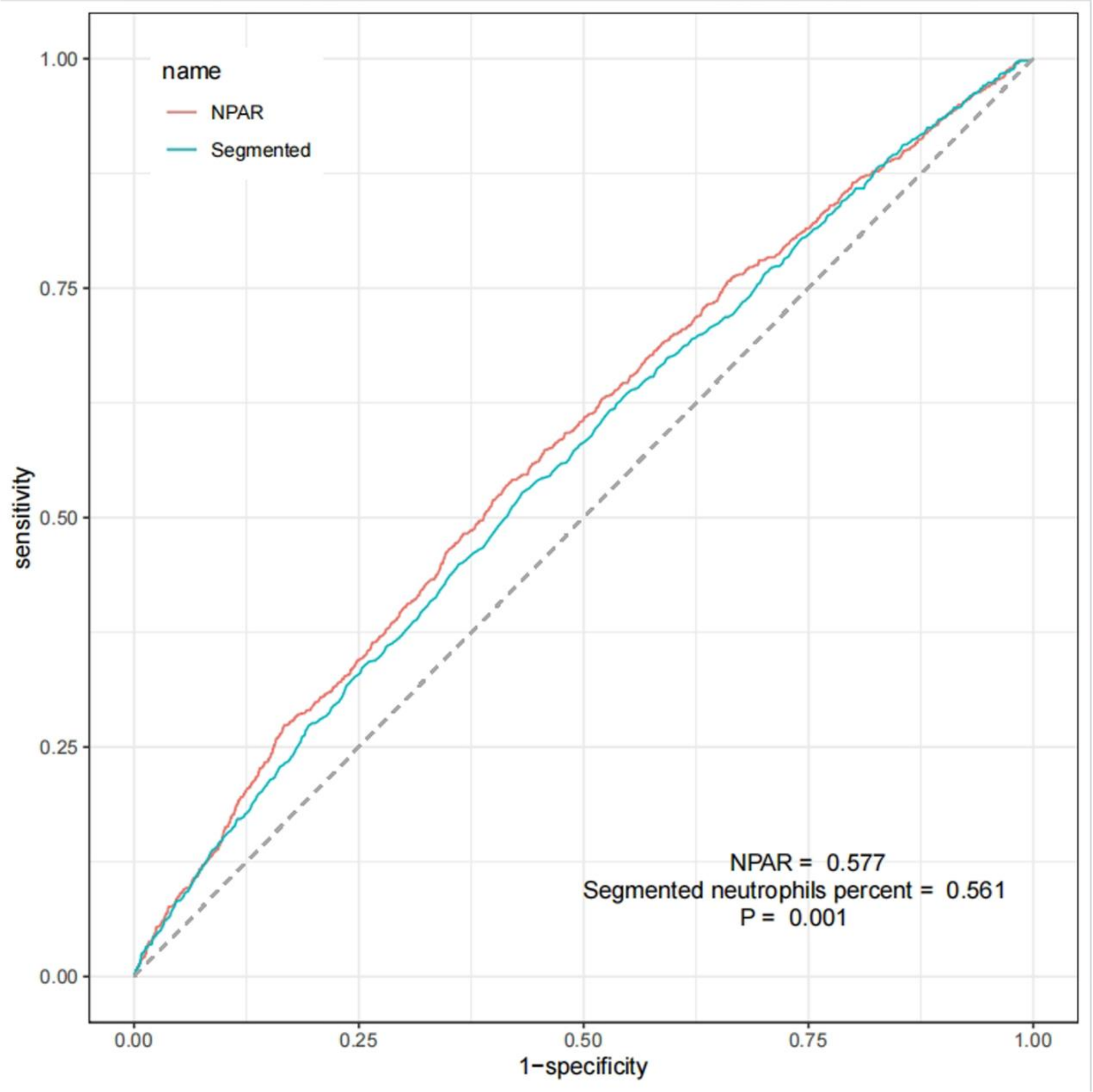

Supplement: Supplementary file 1 [file Datasheet1.pdf]
